# High rates of return to sport after suprascapular nerve decompression: an updated systematic review

**DOI:** 10.1016/j.xrrt.2024.05.007

**Published:** 2024-06-01

**Authors:** Alexis B. Sandler, Matthew E. Wells, Calvin Tran, Rachel Arakawa, Kyle J. Klahs, John P. Scanaliato, Clare K. Green, Carolyn M. Hettrich, John C. Dunn, Adam Adler, Nata Parnes

**Affiliations:** a Texas Tech University Health Sciences Center El Paso, El Paso, TX, USA; b Naval Medical Center Portsmouth, Portsmouth, VA, USA; c George Washington University School of Medicine and Health Sciences, Washington DC, USA; d Midwest Ortho at RUSH University, Chicago, IL, USA; e Carthage Area Hospital, Carthage, NY, USA; f Claxton-Hepburn Medical Center, Ogdensburg, NY, USA

**Keywords:** Rotator cuff, Shoulder arthroscopy, Suprascapular nerve, Suprascapular neuropathy, Spinoglenoid notch, Suprascapular notch

## Abstract

**Background:**

Suprascapular nerve decompression (SSND) remains a controversial procedure. In 2018, Momaya et al published the first systematic review of SSND noting satisfactory outcomes with low rates of complications; however, numerous studies published since have noted no benefit in routinely adding SSND to other arthroscopic surgeries, contributing to existing contention regarding the procedure. The purpose of this study is to provide an updated assessment of outcomes after SSND.

**Methods:**

To conduct this updated systematic review, a search of PubMed (MEDLINE) for relevant studies published prior to January 21, 2023 was conducted. Outcomes including patient-reported clinical outcomes, return to sport, preoperative and postoperative electrodiagnostic testing, and adverse events were collected and pooled for assessment. Studies were eligible for inclusion if they met Momaya et al’s inclusion criteria and/or reported outcomes following SSND at either the suprascapular notch or spinoglenoid notch.

**Results:**

In total, 730 patients from 33 studies were eligible for inclusion. All patient-reported outcome measure scores including American Shoulder Elbow Surgeon Standardized Shoulder Assessment; Constant-Murley score; Disabilities of the Arm, Shoulder, and Hand; Subjective Shoulder Value; University of California–Los Angeles shoulder; and visual analog scale pain scores improved significantly postoperatively, with improvements ranging from 53.5% to 102.6% of preoperative values. Ultimately, 98% (n = 90/92) of patients returned to sport or military duty and 96% of these patients returned at their previous level of activity (n = 48/50) without heterogeneity among rates between studies (*P* = .176, *P* = .238, respectively). Preoperative electrodiagnostic testing was conducted in 93% of patients, and 90% had associated abnormal findings. Continued symptoms were noted among 12% of patients (n = 39/322) with significantly different rates observed between studies. Complications from operative management not limited to SSND occurred in 11% of patients (n = 64/576) and reoperations occurred in 3.3% of patients (n = 15/455).

**Conclusion:**

Suprascapular neuropathy treated with SSND significantly improves patient-reported outcomes and is noninferior to similar procedures without SSND. Appropriate clinical diagnosis of suprascapular neuropathy is required as opposed to a routine adjunct procedure with other arthroscopic shoulder surgery. Ultimately, SSND is associated with high rates of return to sport and relatively low rates of adverse events; however, the risk of continued symptoms and electrodiagnostic test-related complications is an important point on preoperative counseling.

Suprascapular neuropathy was first described nearly a century ago^5^^,^^25^^,^^26^^,^^50^ and, as advancements in diagnostic modalities evolve, has been increasingly recognized as an underlying cause of pain and shoulder dysfunction. Compression of the suprascapular nerve (SSN) can occur secondary to a variety of etiologies including, but not limited to, repetitive overhead activities,^6^^,^^29^^,^^34^^,^^41^ space occupying cysts (sometimes associated with an adjacent superior labral tear),^19^^,^^34^^,^^38^^,^^54^ dysmorphic anatomy,^4^^,^^15^^,^^51^ and retracted rotator cuff tears^2^^,^^12^^,^^34^^,^^44^ and leads to posterior shoulder pain with supraspinatus and/or infraspinatus dysfunction. Chronic injuries can also lead to muscle wasting with underlying fatty infiltration on magnetic resonance imaging. Collectively, the patient’s physical examination, magnetic resonance imaging, and electromyography^7^^,^^37^ findings can facilitate the diagnosis of suprascapular neuropathy. Athletes, especially those participating in overhead sports, are at an increased risk of developing suprascapular neuropathy, with spinoglenoid notch (SGN) compression more common than SSN compression.^9^^,^^48^

The first line of treatment for suprascapular neuropathy involves an initial trial of nonoperative management; however, surgical decompression via suprascapular nerve decompression (SSND) may be indicated in patients with space-occupying lesions or compression from concomitant extrinsic pathologies such as a large rotator cuff tear with retraction.^6^^,^^35^^,^^48^ Recently, interest in SSND has skyrocketed due to conflicting findings in emerging literature: while a systematic review by Momaya et al^37^ demonstrated significant improvement in patient-reported outcome measures (PROMs) and a 92% return to sport (RTS) rate following SSND among 275 patients, recent studies on the role of SSND as an adjunct to rotator cuff repair (RCR) have shown no additional benefit to routine SSND^21^^,^^32^^,^^56^ with a prospective, randomized observational trial by Gerber et al^21^ reporting early termination due to electromyographic complications in 3 patients coupled with an absence of clinical benefit associated with SSND.

The recent revitalization of interest in the role of SSND both independently and as an adjunct to RCR has warranted reassessment after the 2018 systematic review by Momaya et al^37^ given that the number of patients eligible for inclusion has nearly tripled. Subsequently, this study seeks to provide an updated systematic review with the primary objective to determine the PROMs and RTS associated with SSND. The secondary objectives were to characterize current utilization of preoperative electrodiagnostic testing (EDT) as well as rates of adverse events following SSND and concomitant procedures.

## Materials and methods

### Search strategy and study selection

Systematic review and meta-analysis were performed based on the Preferred Reporting Items for Systematic Reviews and Meta-Analyses guidelines. The authors used the search algorithm “suprascapular OR spinoglenoid OR (shoulder and “entrapment neuropathy”) OR (shoulder and “ganglion cyst”) OR (shoulder and “transverse scapular ligament”) based on the search used by Momaya et al^37^ to search the PubMed (MEDLINE) database for all articles published between September 2016 and January 2023 to prevent duplication of articles searched by Momaya et al.^37^ Two investigators (A.B.S. and M.E.W.) independently screened abstracts and performed full-text review. Data collection was performed by 3 independent investigators (A.B.S., C.T., and R.A.) with disputes between data collection discussed and by a separate independent investigator (A.B.S. or M.E.W.).

### Inclusion criteria

To provide a direct and effective update to the systematic review by Momaya et al,^37^ the authors elected to maintain identical inclusion criteria, specifically including studies that reported outcomes following SSND at the SSN or SGN (Table I). To be included, studies had to report at least 1 outcome in the jurisdiction of PROMs, rates of RTS, or EDT results. Articles that did not specify the relevant outcome measures as well as commentaries, editorials, reviews, technical guides, case reports with less than 3 patients, and studies published in languages other than English were excluded.

**Table I:** Inclusion criteria adjusted from Momaya et al.^37^

Inclusion criteria	Exclusion criteria
• Reported results of SSND at SSN and/or SGN • Reported results including at least one of the following: • Patient-reported outcomes • Rates of return to sport rate • EDT • Studies published in English	• Reviews or systematic reviews • Case reports (< 3 patients) • Commentary/editorials • Technical notes • Cadaver studies • Animal studies • Concomitant brachial plexopathy • Concomitant traumatic injuries

*EDT*, electrodiagnostic testing; *SSN*, suprascapular notch; *SSND*, suprascapular nerve decompression; *SGN*, spinoglenoid notch.

### Data analysis and data extraction

Data regarding patient demographics, PROMs, RTS and/or return to military duty, EDT results, and adverse events were collected and pooled for analysis. PROMs included the following: American Shoulder Elbow Surgeons Standardized Shoulder Assessment (ASES) score; Constant-Murley score; Disabilities of the Arm, Shoulder, and Hand (DASH) score; Subjective Shoulder Value (SSV) score; University of California–Los Angeles shoulder score; and the visual analog scale (VAS) pain score. Concomitant procedures were collected based on procedure-specific details specified in the study methodology and were classified as RCR, labral or superior labrum from anterior to posterior lesion repair, subacromial bursectomy/decompression, labral or rotator cuff débridement, acromioplasty, distal clavicle excision, or other. Methodological Index for Non-Randomized Studies (MINORS) criteria were used to assess study quality, although case reports with less than 5 patients and randomized studies did not undergo MINORS criteria calculation given variation in reporting of methodology not thoroughly assessed by the criterion.^47^

### Statistical analysis

Independent 2-tailed *t*-tests were used to evaluate for significant changes based on calculations of weighted means with standard deviations and/or confidence intervals. Chi-squared tests were used to determine significant differences between categorical variables. Multivariate linear regressions were used to assess the impact of concomitant procedures on outcomes following SSND. Statistical significance was set at α less than or equal to 0.05.

## Results

**Figure 1:**
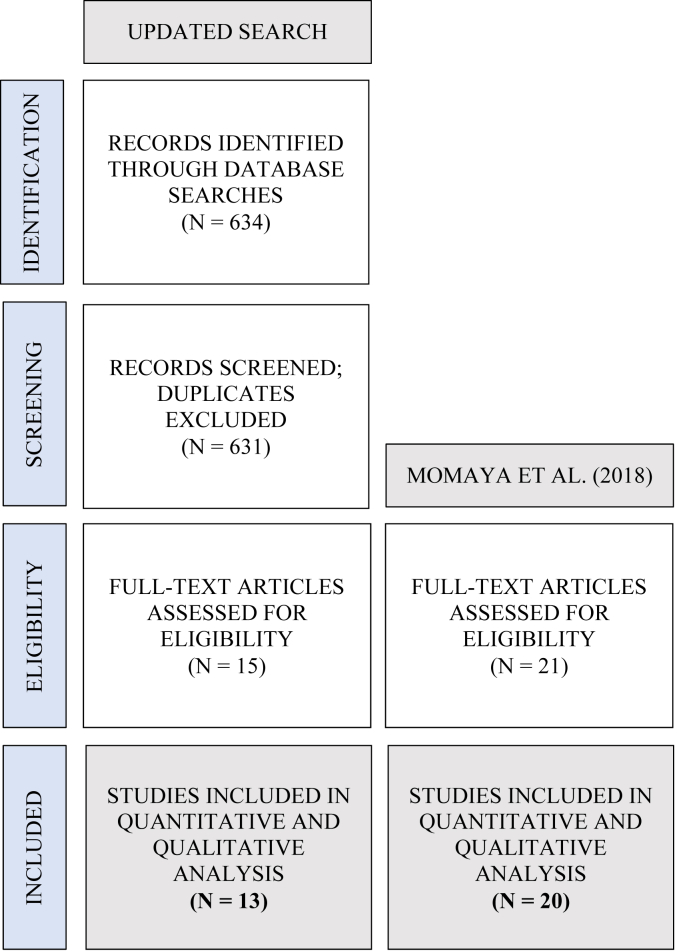
PRISMA flow diagram.

To complete the updated search, an additional 634 abstracts were screened, 15 full-text manuscripts reviewed, and ultimately 13 studies with level I-IV evidence deemed eligible for inclusion (Fig. 1). Quantitative and qualitative data were extracted from the additional 13 studies and combined with data extracted from the 20 articles included in prior systematic review,^37^ totaling data extraction from 33 studies published between 1992 and 2021 (Table II).^1^^,^^3^^,^^8^^,^^10^^,^^12^, ^13^, ^14^^,^^16^, ^17^, ^18^^,^^20^, ^21^, ^22^, ^23^, ^24^^,^^28^^,^^30^^,^^31^^,^^33^^,^^38^, ^39^, ^40^^,^^42^, ^43^, ^44^^,^^46^^,^^52^^,^^54^^,^^55^^,^^57^ Following data extraction, 488 patients from the updated search were pooled with 242 from the Momaya article,^37^ resulting in a total of 730 patients.

**Table II:** Study characteristics.

Study	Journal	Level of evidence	Location of decompression	Technique	Shoulders (n)	Safety concerns warranted termination	MINORS score
Cano-Martínez (2021)^8^	Rev Esp Cir Ortop Traumatol	IV	SSN	Arthroscopic	75	N	11/16
Nolte (2021)^38^	Arthroscopy	IV	SSN/SGN	Arthroscopic	42	N	11/16
Sachinis (2021)^42^	Am J Sports Med	I	SSN	Arthroscopic	37	N	N/A∗
Yang (2021)^57^	BMC Musculoskelet Disord	III	SGN	Arthroscopic	20	N	19/24
Davis (2020)^13^	J Orthop	IV	SSN	Arthroscopic	111	N	13/16
Gerber (2020)^21^	JSES	II	SSN	Arthroscopic	9	Y	N/A∗
Katsuura (2020)^24^	Clin Med Insights Arthritis Musculoskelet Disord	III	SSN	Arthroscopic	100	N	15/24
Feinberg (2019)^17^	Muscle Nerve	II	SSN/SGN	Arthroscopic		N	11/16
Yamakado (2019)^55^	Int Orthop	III	SSN	Arthroscopic	31	N	21/24
Perry (2018)^39^	Mil Med	IV	SSN/SGN	Arthroscopic	7	N	11/16
Tsikouris (2018)^52^	Arthroscopy	III	SSN	Arthroscopic	35	N	17/24
Kim (2017)^25^	Arthroscopy	IV	SGN	Arthroscopic	21	N	11/16
Hashiguchi (2016)^23^	SICOT J	IV	SGN	Arthroscopic	6	N	9/16
Savoie (2016)^44^	Orthop J Sports Med	II	SSN	Arthroscopic	22	N	17/24
Bilsel (2014)^5^	Knee Surg Sports Traumatol Arthrosc	III	SGN	Arthroscopic	16	N	19/24
Leclere (2014)^30^	Arthroscopy	IV	SSN/SGN	Arthroscopic	4	N	N/A†
Arriaza (2013)^3^	Am J Sports Med	IV	SSN	Arthroscopic	4	N	N/A†
Mall (2013)^33^	J Shoulder Elbow Surg	IV	SGN	Open	29	N	11/16
Kim (2012)^26^	J Shoulder Elbow Surg	II	SGN	Arthroscopic	14	N	21/24
Garcia Jr. (2011)^20^	Rev Bras Ortop	IV	SSN	Arthroscopic	10	N	13/16
Pillai (2011)^40^	Clin Orthop Relat Res	IV	SGN	Arthroscopic	6	N	16/24
Shah (2011)^46^	J Shoulder Elbow Surg	IV	SSN/SGN	Arthroscopic	24	N	10/16
Costouros (2007)^12^	Arthroscopy	IV	SSN	Arthroscopic	6	N	13/16
Lafosse (2007)^28^	Arthroscopy	IV	SSN	Arthroscopic	10	N	13/16
Abboud (2006)^1^	Clin Orthop Relat Res	III	SGN	Arthroscopic	16	N	10/16
Westerheide (2006)^54^	Arthroscopy	IV	SGN	Arthroscopic	14	N	11/16
Dramis (2005)^14^	Acta Orthop Belg	IV	SSN	Open	4	N	N/A†
Lichtenberg (2004)^31^	Knee Surg Sports Traumatol Arthrosc	IV	SGN	Arthroscopic	8	N	10/16
Chen (2003)^10^	Arthroscopy	IV	SGN	Arthroscopic	3	N	N/A†
Fabre (1999)^16^	J Bone Joint Surg Br	IV	SSN/SGN	Open	35	N	9/16
Ferretti (1998)^18^	Am J Sports Med	IV	SGN	Open	3	N	N/A†
Sandow (1998)^43^	J Shoulder Elbow Surg	IV	SSN/SGN	Open	5	N	N/A†
Hama (1992)^22^	J Shoulder Elbow Surg	IV	SSN/SGN	Open	3	N	N/A†

*SSN*, suprascapular notch; *SGN*, spinoglenoid notch.

∗ Randomized study.

† Case report with 3-5 studies.

A majority of patients underwent arthroscopic intervention (n = 651, 89.2%) versus open intervention (n = 79, 10.8%). Thirteen studies were specific to SSND performed at the SSN (n = 454 shoulders), while 12 were specific to SSND performed at the SGN (n = 156 shoulders). The remaining 8 studies (n = 128 shoulders) involved a combination of SSND at either the SSN and/or the SGN. The most frequently performed concomitant procedures included RCR (n = 126/638, 19.7%), labral/superior labrum from anterior to posterior lesion repair (n = 98/638, 15.4%), subacromial bursectomy/decompression (n = 49/638, 7.7%), and biceps tenotomy/tenodesis (n = 45/638, 7.1%). Specific concomitant procedures are presented in Table III.

**Table III:** Concomitant procedures.

Author	Rotator cuff repair		Labral/SLAP lesion repair		Subacromial bursectomy/decompression		Biceps tenodesis/Tenotomy		Other	
	N	%	N	%	N	%	N	%	N	Description
Cano-Martínez (2021)^8^	0	0.0%	0	0.0%	0	0.0%	0	0.0%	0	-
Nolte (2021)^38^	0	0.0%	0	0.0%	15	35.7%	8	19.0%	8	Distal clavicle excision (3), rotator cuff healing response (1), os acromial fixation (1), hardware removal (1), axillary nerve decompression (1), musculocutaneous nerve decompression (1)
Sachinis (2021)^42^	18	48.6%	0	0.0%	0	0.0%	18	48.6%	18	Acromioplasty (18)
Yang (2021)^57^	10	50.0%	0	0.0%	0	0.0%	0	0.0%	0	-
Davis (2020)^13^	27	24.3%	5	4.5%	0	0.0%	23	20.7%	0	-
Gerber (2020)^21^	0	0.0%	0	0.0%	0	0.0%	14	74.7%	18	Acromioplasty (14), acromioclavicular joint resection (2), calcium deposit decompression (1), humeral head microfracturing (1)
Katsuura (2020)^24^										
Feinberg (2019)^17^	0	0.0%	8	100.0%	0	0.0%	0	0.0%	0	-
Yamakado (2019)^55^	31	100.0%	0	0.0%	0	0.0%	0	0.0%	0	-
Perry (2018)^39^	0	0.0%	0	0.0%	0	0.0%	7	100.0%	0	-
Tsikouris (2018)^52^	27	77.1%	16	45.7%	31	88.6%	0	0.0%	0	-
Kim (2017)^25^	0	0.0%	0	0.0%	0	0.0%	0	0.0%	0	-
Hashiguchi (2016)^23^	2	33.3%	6	100.0%	0	0.0%	0	0.0%	0	-
Savoie (2016)^44^	22	100.0%	0	0.0%	0	0.0%	0	0.0%	0	-
Bilsel (2014)^5^	0	0.0%	16	100.0%	0	0.0%	0	0.0%	0	-
Leclere (2014)^30^	0	0.0%	0	0.0%	2	50.0%	0	0.0%	0	-
Arriaza (2013)^3^	0	0.0%	0	0.0%	4	100.0%	0	0.0%	0	-
Mall (2013)^33^	0	0.0%	0	0.0%	0	0.0%	0	0.0%	0	-
Kim (2012)^26^	0	0.0%	14	100.0%	0	0.0%	0	0.0%	0	-
Garcia Jr. (2011)^20^	0	0.0%	0	0.0%	9	90.0%	0	0.0%	0	-
Pillai (2011)^40^	0	0.0%	6	100.0%	1	16.7%	0	0.0%	0	-
Shah (2011)^46^	0	0.0%	3	12.5%	0	0.0%	1	4.2%	2	Capsular release (1), distal clavicle excision (1)
Costouros (2007)^12^	6	100.0%	0	0.0%	0	0.0%	0	0.0%	6	Acromioplasty (6)
Lafosse (2007)^28^	0	0.0%	0	0.0%	0	0.0%	0	0.0%	3	Distal clavicle excision (3)
Abboud (2006)^1^	0	0.0%	9	56.3%	0	0.0%	0	0.0%	0	-
Westerheide (2006)^54^	0	0.0%	7	50.0%	2	14.3%	0	0.0%	1	Distal clavicle excision (1)
Dramis (2005)^14^	0	0.0%	0	0.0%	0	0.0%	0	0.0%	0	-
Lichtenberg (2004)^31^	0	0.0%	5	62.5%	0	0.0%	0	0.0%	2	Capsular release (2)
Chen (2003)^10^	0	0.0%	3	100.0%	0	0.0%	0	0.0%	0	-
Fabre (1999)^16^	1	2.9%	0	0.0%	0	0.0%	0	0.0%	0	-
Ferretti (1998)^18^	0	0.0%	0	0.0%	0	0.0%	0	0.0%	1	Shaved scapular spine (1)
Sandow (1998)^43^	0	0.0%	0	0.0%	0	0.0%	0	0.0%	0	-
Hama (1992)^22^	0	0.0%	0	0.0%	0	0.0%	0	0.0%	2	Shaved scapular spine (2)
Total	126	19.7%	98	15.4%	49	7.7%	45	7.1%	61	-

*SLAP*, superior labrum from anterior to posterior.

### Patient-reported outcome measures (PROMs)

Significant postoperative improvements in PROMs were observed among all PROM scores with increases in ASES, Constant-Murley score, SSV, and University of California–Los Angeles scores with concurrent decreases in DASH and VAS scores (Table IV). Changes in preoperative to postoperative PROM scores ranged from 53.5% to 102.6% improvement. Multiple linear regression revealed no statistically significant effects of rates of associated RCR, labral repair, and biceps tenodesis/tenotomy on postoperative VAS scores (*P* = .583) with insufficient data available to test other PROM measures.

**Table IV:** Patient-reported outcome measure (PROM) scores.

Score	Mean (SD)		Included studies (patients), n	PROM change, points (95% CI)	PROM change, %	T-statistic	*P* value
ASES	Preoperative	47.5 (2.0)	113 (6)	+25.4 (21.5-29.4)	+53.5%	+12.8	<.001
	Postoperative	72.9 (16.5)	114 (6)				
Constant	Preoperative	54.7 (18.0)	208 (9)	+29.6 (27.1-32.2)	+54.1%	+22.8	<.001
	Postoperative	84.3 (6.9)	222 (9)				
DASH	Preoperative	67.8 (21.0)	95 (3)	−50.3 (46.0-54.7)	−74.2%	−22.9	<.001
	Postoperative	17.5 (4.3)	95 (3)				
SSV	Preoperative	48.4 (12.0)	58 (4)	+32.0 (27.3-36.6)	+64.8%	+13.6	<.001
	Postoperative	80.4 (13.3)	58 (4)				
UCLA	Preoperative	15.3 (4.6)	131 (7)	+15.7 (14.8-16.6)	+102.6%	+35.2	<.001
	Postoperative	31.0 (2.3)	131 (7)				
VAS	Preoperative	6.8 (1.3)	481 (15)	−4.2 (4.0-4.4)	−61.8%	−39.4	<.001
	Postoperative	2.6 (1.9)	463 (13)				

*ASES*, American Shoulder Elbow Surgeons Standardized Shoulder Assessment score; *CI*, confidence interval; *Constant*, Constant-Murley score; *DASH*, Disabilities of the Arm, Shoulder, and Hand score; *SSV*, Subjective Shoulder Value score; *UCLA*, University of California–Los Angeles shoulder score; *VAS*, visual analog scale pain score; *PROM*, patient-reported outcome measures; *SD*, standard deviation.

### Return to sport

The vast majority of patients were able to RTS or military duty (n = 90/92, 98%, Table V) without significant differences observed among rates between studies (X^2^ [10, n = 92] = 2.658, *P* = .988). Furthermore, 96% (n = 48/50) were able to RTS or duty at their previous level of function without significant differences observed among rates between studies (X^2^ [8, n = 50] = 11.48, *P* = .176). The only specified athlete unable to RTS at previous level of play was a javelin thrower reported by Fabre et al.^16^ On a broader level, 94% (n = 123/131) of patients reported return to function to include sport participation, military duty, work, and activity following SSND without significant differences observed among rates between studies (X^2^ [11, n = 131] = 13.90, *P* = .238). Pooled specific sports and military occupations demonstrated the largest cohort to be volleyball players, at 25 athletes (Table VI).

**Table V:** Return to sport/military duty by study.

Author	Total population, athletes or Military, n	Rates of return to sport/Duty, %	Rates of return to sport/Duty at prior level, %	Specific sports activities
Perry (2018)^39^	7	100%	-	Navy sea, air, and land (2), Navy diver (1), AD Navy other (3), AD Marine (1)
Tsikouris (2018)^52^	35	97.1%	-	Volleyball (33), water polo (8), weightlifting (9), javelin throw (6)
Arriaza (2013)^3^	4	100%	100%	Swimming (4)
Kim (2012)^26^	14	92.9%	92.9%	Unspecified
Westerheide (2006)^54^	10	100%	100%	Unspecified
Dramis (2005)^14^	4	100%	100%	Volleyball (4)
Chen (2003)^10^	3	100%	100%	Modern dance (1), softball (1), tennis (1)
Fabre (1999)^16^	4	100%	75%	Javelin throw (1), swimmer (2), volleyball (1)
Ferretti (1998)^18^	3	100%	100%	Volleyball (3)
Sandow (1998)^43^	5	100%	100%	Volleyball (5)
Hama (1992)^22^	3	100%	100%	Volleyball (2), tennis (1)
Total	92	97.8% (n = 90/92)	96% (n = 48/50)	

*AD*, Active duty.

Patients could report more than 1 sport.

**Table VI:** Specific pooled sports and military occupations.

Activity	N
Return to sport	111
Volleyball	52
Weightlifting	9
Water polo	8
Javelin throw	7
Swimming	6
Tennis	3
Dance	1
Softball	1
Unspecified	24
Military occupations	7
Active-duty Navy, other	3
Navy sea, air, and land	2
Navy diver	1
Active-duty Marine, other	1

### Electrodiagnostic testing (EDT)

In total, 25 studies reported preoperative EDT (n = 678, 92.9%), while only 13 reported postoperative EDT (n = 361, 49.5%). Of the pooled patients undergoing both preoperative and postoperative EDT irrespective of whether it was a study requirement, 90.0% (n = 190/211) were noted to have abnormal findings on preoperative EDT. Of these, 31.6% (n = 60/190) continued to have some degree of EDT abnormality on latest recorded follow-up in the study period.

### Adverse events

Continued symptoms were noted among 12% of patients (n = 39/322) with significantly different rates observed between studies (X^2^ [12, 322] = 81.45, *P* < .001). In total, 29 patients presented with continued pain or discomfort, 5 with weakness or atrophy, 3 with EDT-related complications, and 2 with continued dissatisfaction with ultimate functional outcomes. The 3 patients with EDT-related complications reported by Gerber et al^21^ warranted premature termination of the study. Complications from operative management not limited to SSND occurred in 11% of patients (n = 64/576) and reoperations in 3.3% of patients (n = 15/455, Table VII).

**Table VII:** Adverse events.

	N (%)
Suprascapular nerve decompression complications	
Continued pain or discomfort	29 (9.0%)
Muscle weakness or atrophy	5 (1.6%)
Dissatisfaction with outcomes	2 (<1%)
Associated procedure complications	
Rotator cuff retear	16 (2.8%)
Sympathetic dystrophy	14 (2.4%)
Worsened spine-mediated radicular pain	11 (1.9%)
Repair failure	4 (0.7%)
Shoulder arthritis	4 (0.7%)
Superficial soft tissue infection	4 (0.7%)
Healing failure	3 (0.5%)
Adhesive capsulitis	3 (0.5%)
Transverse skin thickening	2 (0.3%)
Musculocutaneous nerve injury	1 (0.2%)
Reoperation	
Revision of index procedure	9
Anatomic or reverse total shoulder arthroplasty	3
Hemiarthroplasty	1
Biceps tenodesis	1
Spine surgery for radicular pain	1

The majority of complications and revisions were associated with concomitant procedures rather than isolated SSND (Table III). Similarly, multiple linear regression investigating the effect of concomitant RCR, labral repair, and biceps tenodesis/tenotomy on outcomes including RTS and persistent symptoms demonstrates statistically significant intercepts at 1.00 (0.98-1.02, *P* < .001) and 0.13 (confidence interval = 0.39-0.22, *P* = .007), respectively, indicating baseline rates of RTS at 100% and persistent symptom rates of 13% without concomitant procedures. While there were insignificant data to compare RTS rates with associated procedures in SSND performed at the SSN versus SGN, there were no significant trends noted in persistent symptoms for SSND performed at either the SSN (*P* = .848) or the SGN (*P* = .729).

## Discussion

This expanded systematic review demonstrates that SSND is associated with statistically significant improvements in all PROM scores and that the vast majority of patients returned to sport or military duty with low rates of adverse events directly related to SSND. Additionally, a vast majority of patients present with preoperative EDT abnormalities prior to SSND; however, up to 30% will continue to have postoperative abnormalities despite improvements in PROMs. Collectively, this systematic review supports SSND as a safe and effective surgical treatment for suprascapular neuropathy with high rates of RTS and military duty, which is especially important given the higher rate of suprascapular neuropathy observed among these populations.^9^^,^^48^

### Patient-reported outcome measures (PROMs)

In this study, all 6 measured PROMs improved significantly following SSND. While there is scant literature to confirm the minimal clinically important difference values specific to SSND, assessing the 25.4-point improvement in ASES following SSND as compared to the 15.4 (standard deviation = 5.7) point ASES threshold for other shoulder procedures indicates substantial clinical improvement. In comparing the substantial clinical benefit and patient acceptable symptom state thresholds defined for subacromial impingement, an 11-point improvement in DASH scores and a final VAS pain score of 3.0 or less indicates clinical benefit.^49^ Promisingly, improvements in PROMs observed in this study are consistent with existing literature, with Momaya et al and Memon et al both reporting significant improvements in VAS pain scores in addition to improvements in postoperative strength testing.^36^^,^^37^ In appropriately indicated patients, these results suggest that SSND can be expected to improve measures of patient pain and function postoperatively, although future research establishing minimal clinically important difference, substantial clinical benefit, and patient acceptable symptom state thresholds specific to SSND will offer value in understanding a targeted threshold for clinical improvements associated with this procedure.

### Return to sport

Overhead athletes are at risk for various shoulder injuries^45^ and compose the predominant athletic population at risk for developing suprascapular neuropathy.^29^^,^^43^^,^^48^ While the reason for suprascapular neuropathy in this population is not fully understood, it has been theorized that the high incidence of labral tears generates paralabral cysts that lead to the development of suprascapular neuropathy.^36^ In this study, 98% of patients were able to RTS or military duty with 96% of those patients able to RTS or duty at their previous functional level. Similar findings have been reported in other large reviews, with a notably high (>85%) return to play.^36^^,^^37^ The high rate of RTS among this patient cohort supports the assertion that SSND offers benefits to the athlete population and does not appear to prevent return to activity. However, the functional improvements afforded by SSND appear to be limited to patients with suprascapular neuropathy, as multiple recent studies have shown that SSND offers no benefit as a routine adjunct to RCR.^21^^,^^32^^,^^56^ Subsequently, identifying patients with symptomatic suprascapular neuropathy or SSN/SGN compression becomes critical in indicating appropriate patients for SSND rather than including SSND in all RCR procedures by default.

### Electrodiagnostic testing (EDT)

Based on the results of this present study, it is prudent that patients with physical examination and advanced imaging findings suggestive of suprascapular neuropathy undergo neurodiagnostic testing to aid in the diagnosis and establish a pretreatment baseline.^48^ As discussed, there are many potential causes for suprascapular neuropathy with labral tear-associated cysts and retracted rotator cuff tears being among the most common. In the present study, 93% of patients underwent preoperative EDT and 90% of patients with preoperative EDT had associated abnormal findings. In their direct comparison, Tsikouris et al found that high-level athletes with rotator cuff tears or glenoid labral cysts with abnormal preoperative suprascapular EDT had superior clinical outcomes when treated with combined arthroscopic repair/cyst decompression in combination with SSND as compared to the indexed arthroscopic procedure alone.^52^ Given that routine SSND for RCR appears to lack substantial benefits, achieving a preoperative suprascapular neuropathy diagnosis offers value in establishing which patients will benefit from SSND.^21^^,^^32^^,^^56^ Based on the findings of the present study, the authors believe that iatrogenic injury to the nerve and the associated complication profile during the procedure should only be risked if a preoperative suprascapular neuropathy is clinically diagnosed.

Importantly, however, not all patients with rotator cuff tears or paralabral cysts will have abnormal EDT findings of suprascapular neuropathy. Collin et al prospectively performed EDT on all their patients undergoing RCR and found only 2% showed abnormal EDT findings.^11^ Various other studies have reported higher prevalence rates; however, it is important to note these higher rates were more common in massive rotator cuff tears.^7^^,^^12^^,^^27^^,^^53^ Gerber et al^21^ performed a prospective, randomized control trial on combining SSND in RCR for patients without preoperative EDT abnormalities but ultimately terminated the study early as emerging findings explicitly showed no benefit for the adjunct nerve decompression in this setting. Ultimately, the high rate of successful PROM achievement and RTS in a population with 90% abnormal preoperative EDT described in this study suggests that SSND appears to have added benefits when indicated as a result of abnormal preoperative EDT findings.

### Adverse events

In this review, approximately 12% of patients reported continued pain or discomfort in the postoperative setting, while 11% experienced complications and 3.3% required subsequent surgery. It is our opinion, based on the nature of included studies, that the rates of adverse events are undoubtedly overestimates given the high rate of concomitant procedures associated with SSND coupled with a common lack of differentiation between SSND-related adverse events from other procedural adverse events. This is reinforced by the significant variability among complication and reoperation rates between included studies. When limited to rates of persistent symptoms and RTS associated with RCR, labral repair, and biceps tenodesis/tenotomy rates, the statistically significant estimated rate of these persistent symptoms was 13% without evidence of statistically significant changes based on the 3 associated procedures, likely explaining the overall rates of adverse events in this article that appear higher than similar reviews.^36^^,^^37^ However, there was significant heterogeneity between the included studies that should be interpreted accordingly. Importantly, after accounting for continued pain or discomfort, the adverse events rates were notably similar.

### Limitations

As is the nature of any systematic review, the conclusions drawn in the present study are limited by the evidence reported by articles eligible for inclusion. PROMs selected for inclusion varied significantly between studies and, other than VAS, were not reported widely enough to determine how concomitant procedures impact final PROM scores. Additionally, rates of adverse events included those specific to SSND as well as other concomitant procedures, thereby overestimating complication and reoperation rates from SSND directly. Additionally, this study did not attempt to compare open versus arthroscopic SSND and faced limitations in separately assessing outcomes after SSND at the SSN versus the SGN. Overall, there is a paucity of prospective comparative studies in the use of EDT and open verses arthroscopic management and the generalizability of this article may be limited in centers without the use of EDT or for surgeons with limited training in surgical techniques of SSND.

## Conclusion

Suprascapular neuropathy treated with SSND significantly improves patient-reported outcomes and is noninferior to similar procedures without SSND. Appropriate clinical diagnosis of suprascapular neuropathy is required as opposed to a routine adjunct procedure with other arthroscopic shoulder surgery. Ultimately, SSND is associated with high rates of RTS and relatively low rates of adverse events; however, the risk of continued symptoms and electrodiagnostic test-related complications is an important point on preoperative counseling.

## Disclaimers:

Funding: This research did not receive any specific grant from funding agencies in the public, commercial, or not-for-profit sectors.

Conflicts of interest: Each author certifies that they have no commercial associations (e.g., consultancies, stock ownership, equity interest, patent/licensing arrangements, etc.) that might pose a conflict of interest in connection with the submitted article.
